# Editorial: Ancient crop varieties based food intake: impact on nutritional quality, human health and environmental sustainability

**DOI:** 10.3389/fnut.2023.1211358

**Published:** 2023-06-28

**Authors:** Emanuela Simonetti, Monica Dinu, Francesco Sofi, Giovanni Dinelli, Sara Bosi

**Affiliations:** ^1^Kamut Enterprises of Europe, Bologna, Italy; ^2^Department of Experimental and Clinical Medicine, University of Florence, Florence, Italy; ^3^Department of Agricultural and Food Sciences (DISTAL), Alma Mater Studiorum - University of Bologna, Bologna, Italy

**Keywords:** ancient crop varieties, modern crop varieties, functional foods, dietary quality, human health, biodiversity, environmental sustainability

Is the food we eat what our grandparents ate? The obvious answer would be no. However, it is not just a matter of having adopted modern food processing technologies or making extensive use of chemical additives to make food more palatable and keep it longer. Considering plant-based food, it is the varieties of the leading food crops that have changed as well as the agriculture systems that produce them.

Over the centuries, farmers have grown seed that they selected from the wild and adapted to a specific environment and climate. After the Second World War, chemistry forcefully entered agriculture and the fundamental concept of the plant-environment relationship was dramatically altered: it was no longer the plant that adapts to the environment, but it is the environment that must adapt to the plant. The so-called Green Revolution starting from the second part of the last century led to the massive use of technological inputs and to the development of new plant varieties with higher yields and improved technological qualities to produce a cheap and plentiful supply of food. The ancient crop varieties, which were traditionally cultivated for millennia, were progressively replaced with the introduction of few modern crop varieties cultivated on a large scale as monocultures with a consequent loss of biodiversity.

Besides the environmental impact caused by the introduction of modern crop varieties, what has been their impact on human health?

There is growing scientific evidence suggesting a possible role of modern plant varieties in the recent increase of the number of people suffering from food intolerances because they are so different from the original traditional varieties. Moreover, several studies evidenced the unique nutraceutical, phytochemical and sensorial value of ancient crop varieties, suggesting their interesting potential to be part of a healthier and high nutritional diet. For example, ancient wheat varieties have been recently associated to beneficial effects on cardiometabolic risk profile and on pro-inflammatory/antioxidant parameters (1).

However, the debate on the alleged healthier potential of ancient crop varieties compared to modern varieties is still open; hence the need for more studies on the nutritive value and the phytochemical profile of ancient crop varieties and their impact on human health compared to modern crop varieties.

In this context, this Research Topic aims to highlight the significance in human nutrition of rediscovering those ancient crop varieties which have not been modified by modern breeding technologies.

Baldi et al. evaluated the impact of consuming pasta made with ancient wheat, compared to pasta made with modern wheat, on gut microbiota composition and its metabolites' production. Gut microbiota is involved in several metabolic processes and its composition is influenced by general food quality. Both interventions were able to significantly modify the gut microbiota composition at the genus level, with reported increased number of species associated with diseases like cardiovascular, chronic intestinal inflammation, and Parkinson's disease only after the modern wheat pasta diet. Regarding the metabolite production, the intake of ancient wheat pasta had a greater beneficial impact on anti-inflammatory short-chain fatty acids (SCFAs).

Turning to a minor crop, Weng et al. presented a review about Adlay (*Coix lacryma-jobi* L.), an ancient cereal crop that originated in Asia and Africa and is closely related to maize and sorghum. High in protein, polyphenols, vitamins and minerals, and with other bioactive components such as the polysaccharide coixan and several functional lipids, coix seeds show many pharmacological effects, including antioxidant, anti-inflammatory, anti-cancer, hypoglycemic and lipid-lowering effects. Reviewing the studies on the functional ingredients of Adlay, the authors promoted its use for the development of functional healthy food.

Traditionally, farmers made seed selections also to obtain plants rich in flavors and aromas and, since these attributes are particularly influenced by secondary plant metabolites such as polyphenols, the result was also an increased nutritional quality. The modern selections have focused on increasing the total crop production and technological quality while less attention has been paid to other aspects related to the nutritional and organoleptic properties.

With the aim of exploring the potential of wheat landraces, Frankin et al. analyzed the dough quality, the rheology, the aroma and the taste of flours and breads prepared with a collection of Israeli and Palestinian durum and bread wheat landraces. In comparison to modern wheat varieties, wheat landraces were characterized by unique flavors which were particularly appreciated by consumers and well-appropriate for baking. The authors supported the development of high nutritious food based on wheat landraces for their reintroduction in the market and addressed the importance of conservation of wheat landrace germplasm.

Recent studies evidenced that ancient crop varieties are rich in essential nutrients with antioxidant and anti-inflammatory properties. Among these, vitamin C is a strong antioxidant that the human body cannot synthesize by itself but must be introduced with the diet. Cai et al. investigated the relationship between vitamin C intake and human telomere length on 7,094 participants and evidenced a positive correlation. This has important implications in clinical practice.

Wendell Berry said: “Eating is an agricultural act.” By choosing what we eat, we also choose the type of agriculture that produces our food.

Ancient crop varieties don't need chemicals to grow, are particularly suitable for organic growing systems, making them more sustainable for the environment. Moreover, the ancient crop varieties show peculiar nutritional and health properties, and, last but not least, sensorial qualities (2).

Even if the number of studies is still limited and definitive conclusions can't be drawn, we hope the present Research Topic can offer new insights in this area and promote interest in further research required for the understanding of the nutritional and health potential of ancient crop varieties.

## Author contributions

All authors listed have made a substantial, direct, and intellectual contribution to the work and approved it for publication.
